# Recent Advances in the Utilization of Dietary-Derived Exosome-like Nanoparticles in Inflammatory Bowel Diseases

**DOI:** 10.3390/foods15030463

**Published:** 2026-01-29

**Authors:** Zhu Lin, Yanhan Cui, Jing Wang, Yonghui Yu, Chen Tan

**Affiliations:** Key Laboratory of Geriatric Nutrition and Health (Beijing Technology and Business University), Ministry of Education, China-Canada Joint Lab of Food Nutrition and Health (Beijing), School of Food and Health, Beijing Technology and Business University (BTBU), Beijing 100048, China; linzhu202302@163.com (Z.L.); 18637394090@163.com (Y.C.); wangjing@th.btbu.edu.cn (J.W.); yonghuiwh@btbu.edu.cn (Y.Y.)

**Keywords:** exosome-like nanoparticles, inflammatory bowel disease, intestinal microbiota, delivery system

## Abstract

Inflammatory bowel disease (IBD) is a group of chronic autoimmune diseases, including Crohn’s disease and ulcerative colitis. The incidence of IBD has been increasing in newly industrialized countries, whereas current conventional therapeutic medications are ineffective and have unavoidable side effects. In recent years, exosome-like nanoparticles (ELNs) isolated from natural dietary ingredients have played a positive role in the treatment of IBD. These vesicles can exert therapeutic effects on IBD through modulating inflammation-related cytokines, repairing the intestinal barrier, and regulating the intestinal microbiota. In this study, we outline the physiological functions and applications of plant-and animal-derived of ELNs in IBD. Particular emphasis is given to the therapeutic mechanism and efficacy of these ELNs Additionally, the modification of ELNs and loading bioactive compounds in ELNs for improved effects on IBD are discussed. Finally, the prospects and challenges of the dietary-derived ELNs are proposed.

## 1. Introduction

Inflammatory bowel disease (IBD) is a group of chronic intestinal disorders that usually includes Crohn’s disease and ulcerative colitis [1]. It is considered as a global disease with a generally high prevalence in Western countries. In recent years, the prevalence has been increasing annually in the newly industrialized countries of Asia, such as China and India [2]. Although the exact pathogenesis of IBD remains unknown, research studies have shown that it is associated with a number of environmental factors, such as living hygiene conditions, vaccination, use of antibiotics, and dietary intake [3]. Gut microbiota dysbiosis may be a trigger for IBD-initiating events [4]. This is because the reduction in the abundance and diversity of the microbiota could dysregulate gut ecology and make the host more susceptible to pathogen colonization [5]. Dysbiosis of the intestinal flora characterizes IBD and is associated with impaired epithelial barrier function and inflammation [6]. In addition, genetic studies and mouse models have highlighted the importance of genetic factors relative to the defects in the epithelial barrier, neutrophil, regulatory T-cells, T-cells and/or B-cells, and interleukin (IL)-10 signaling, or other hyperinflammatory and autoinflammatory disorders, and the interaction of genetic susceptibility genes with microbial and environmental factors to promote the development of colon-related diseases [7].

The conventional treatment for IBD is the use of medications, including aminosalicylates, corticosteroids, thiopurines, methotrexate, and anti-tumor necrosis factor drugs [8]. However, the inappropriate use of medications, including under-administration, over-administration, or delayed administration of medications, may occur in conventional treatment, which usually results in a delay in the disease [8]. There are also gastrointestinal and hepatic adverse effects including nausea, diarrhea, vomiting, abdominal pain, and fatigue when using drugs for long-term treatment. Potential side effects of thiopurine drugs are nodular regenerative hyperplasia, and hepatotoxicity is unavoidable with the use of methotrexate in the treatment of IBD in addition to the occurrence of myelosuppression and pulmonary fibrosis [9]. Based on this, there is a clinical need to find new, safe, and effective strategies for the prevention or treatment of IBD. Dietary factors are strongly associated with the incidence and progression of IBD [6]. Functional foods can be used to protect gut microbes and intestinal epithelial cells, modulate the microbiota, and produce a number of beneficial substances [10]. An example is the intake of fiber-rich prebiotics from dietary sources, which can be fermented and increased in abundance by the host microbiota, thus leading to a positive effect on the gut [11]. Plant-derived secondary metabolites such as phenolic compounds, terpenoids, alkaloids, and oligosaccharides can modulate enzyme activities, inhibit inflammatory transcription factors, alleviate oxidative stress, and reduce the secretion of pro-inflammatory cytokines, which are potential therapeutic avenues for IBD [12,13].

In recent years, ELNs isolated from dietary sources such as fruits, vegetables, herbal, and dairy products have shown disease-mitigating and gut microbiota-regulating effects [14]. ELNs are a subset of extracellular vesicles that contain proteins, amino acids, lipids, and metabolites. ELNs mediate intercellular communication and play an important role in disease diagnosis and therapy [15]. ELNs derived from edible plants have bioactivity or pharmacological functions similar to those of the original plants, while at the same time, due to their nano-size, they have higher bioavailability and barrier permeability, and exert anti-inflammatory and immunomodulatory functions in a variety of diseases, including cancer, colitis, and inflammation-related diseases [16]. Plant-derived ELNs are also excellent delivery vehicles for functional compounds due to their low immunogenicity, high cellular uptake, great gastrointestinal stability, and targeting ability. Additionally, the lipid bilayer of the ELNs can protect the cargo from being broken down by proteases and nucleases [17].

**Figure 1:**
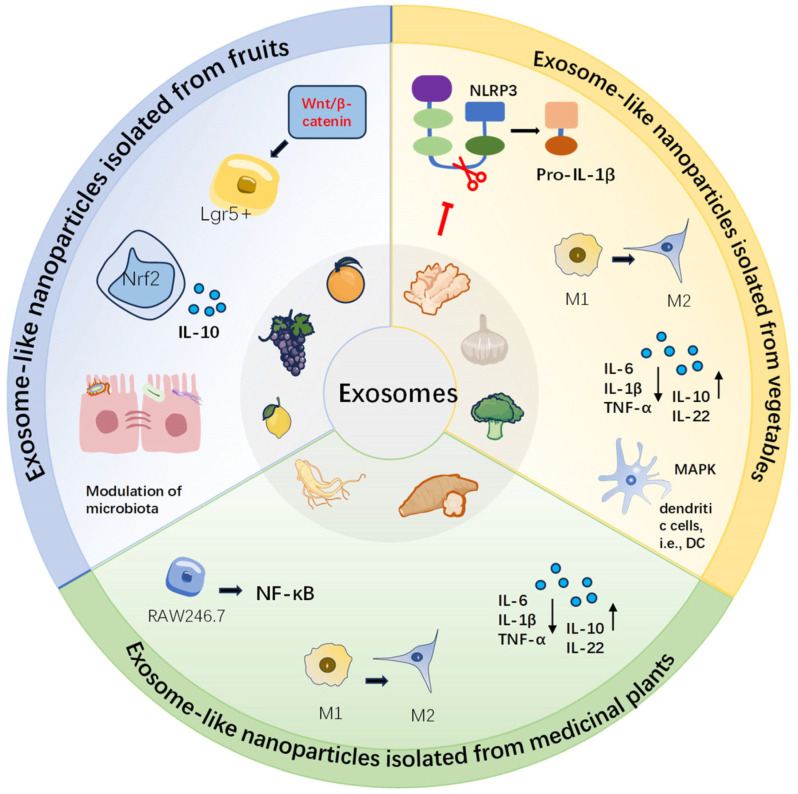
Schematic of ELNs from fruits, vegetables, and medicinal plants and the key modulating mechanisms on intestinal homeostasis and therapeutic effects in IBD.

Although there are some reviews on dietary ELNs and IBD, most of them discuss the single therapeutic mechanisms, and the dual role of ELNs as “therapeutic agents + bioactive component carriers” is rarely integrated [18,19,20]. To address these gaps, this review provides a comprehensive analysis of both plant- and animal-derived dietary ELNs, systematically elucidates their multi-target therapeutic mechanisms, and highlights recent advances in ELN modification and bioactive loading for enhanced efficacy (Figure 1). We aim to offer key insights for the design and clinical translation of dietary ELN-based IBD treatment strategies. At the same time, the current prospects and challenges in this field are discussed.

## 2. ELNs from Edible Plant Sources for IBD Treatment

Edible plants contain vegetables, fruits, and traditional herbs that often contain bioactive compounds such as polyphenols, polysaccharides, alkaloids, and terpenoids. Consumption of plant foods can interact with gut microorganisms, thereby affecting the microbiota axis involved in organ diseases [21]. ELNs from edible plant sources have also shown health benefits such as modulation of the gut microbiome and homeostasis, promotion of intestinal tissue regeneration, and anti-inflammatory and anti-tumor effects [22]. Table 1 summarizes the core characteristics and mechanism of action of ELNs from edible plant sources.

### 2.1. ELNs Isolated from Vegetables

Ginger, a perennial herb whose rhizome is one of the most popular edible spices, contains multiple bioactive components, including volatile oils, gingerol analogs, steroids, and more than 160 other biologically active compounds. Studies have shown that these components have a variety of effects including gastrointestinal protection, anti-cancer properties, and obesity prevention [48]. Ginger also demonstrates broad anti-inflammatory activity. Studies suggest that ginger-derived ELNs (GELNs) can inhibit NOD-like receptor family pyrin domain containing 3 (NLRP3) inflammatory vesicle activation in primary macrophages and may strongly inhibit the release of cytokines IL-1β and IL-18, caspase-1 autocleavage, and pyroptotic cell death [23]. These nanoparticles are reported to be internalized by intestinal cells, potentially counteracting lipopolysaccharide (LPS)-induced inflammation by down-regulating nuclear factor kappa B (NF-κβ) and inflammatory cytokine expression [24]. In a dextran sulfate sodium (DSS)-induced mouse model of colitis, the oral administration of GELNs effectively targeted colonic tissues, increased survival and proliferation of intestinal epithelial cells, decreased pro-inflammatory cytokines (TNF-α, IL-6, and IL-1β), and reduced anti-inflammatory cytokines (IL-10 and IL-22), which facilitated wound healing of the intestinal mucosa [25]. In another work, GELNs were obtained by a commercial ELNs isolation kit and differential centrifugation. Results from that study showed that these nanoparticles induced M2 macrophage activation to alleviate the intestinal inflammatory response. This was proposed to be attributed to the fact that the ELNs-specific miRNA, Osa-miR164d, specifically and directly regulated TAB1 expression in host cells, which regulated macrophage polarization by down-regulating NF-κB expression [26]. GELNs have also been reported to improve colitis through gut-microbiome modulation. According to one mechanistic study, GELNs were preferentially absorbed by Lactobacillaceae in a lipid-dependent manner. Their microRNA content (e.g., mdo-miR7267-3p) targeted *Lactobacillus rhamnosus* monooxygenase *ycnE* genes, leading to higher production of indole-3-carboxaldehyde (I3A). This metabolite subsequently enhanced IL-22 secretion, a cytokine critical for intestinal barrier function, thereby ameliorating colitis [27].

Garlic-derived ELNs are also reported to inhibit pathways downstream of NLRP3 inflammatory vesicle activation [28]. They exerted a protective effect in DSS-induced colitis in mice, ameliorating bloody diarrhea, normalizing pro-inflammatory cytokine production, and preventing colonic barrier damage. This was mainly attributed to peu-MIR2916-p3 in garlic ELNs, which was found to specifically promote the growth of the intestinal commensal bacterium *Bacteroides immitis* [29]. Another work showed that Han-miR3630-5p in garlic-derived ELNs bound to the 3′ untranslated region of toll-like receptor 4 (TLR4) inhibited the TLR4/MyD88/NF-κB signaling pathway, and therefore suggested a potential to protect the colon from DSS-induced injury [30].

Shiitake mushroom-derived ELNs were shown to significantly inhibit NLRP3 inflammatory vesicle activation in primary macrophages and also inhibited IL-6 secretion, as well as protein and mRNA levels of the *Il1b* gene [49]. Broccoli-derived nanoparticles can target adenosine monophosphate-activated protein kinase (AMPK) in dendritic cells to regulate intestinal immune homeostasis, due to the function of the radish sulfide in these nanoparticles [31]. Oral administration of kidney bean-derived ELNs ameliorated obesity in rats, mainly by modulating the composition of the intestinal flora, increasing the distribution of beneficial bacteria, and increasing the levels of short-chain fatty acid metabolites. This may be due to the fact that the functional components of kidney beans are rich in short-chain fatty acids (SCFA) [32].

### 2.2. ELNs Isolated from Fruits

Edible fruits contain very high levels of beneficial components such as dietary fiber, vitamins, polyphenols, minerals, and bioactive peptides. Recent studies have shown that these compounds isolated from fruits can exert positive effects on IBD by exhibiting anti-inflammatory, immunomodulatory, and antioxidant properties [50]. Overall, ELNs derived from fruits exhibit some common advantages, such as good biocompatibility, oral stability, and the ability to alleviate colitis by regulating key signaling pathways, gut microbiota, and the epithelial barrier. However, the therapeutic focus and mechanistic pathways of ELNs differ across different fruit species.

Grapefruit-derived ELNs are enriched in phosphatidylethanolamine and phosphatidylcholine that have antioxidant and anti-inflammatory properties. One study reported that they can induce nuclear translocation of macrophage Nrf2 and intestinal Wnt/TCF4 activation in IL-10 knockout mice with spontaneous colitis upon oral administration. [33]. In a DSS-induced mouse model of colitis, the same research indicated that grapefruit-derived ELNs ameliorated colitis by inhibiting IL-1β and TNF-α production in intestinal macrophages and up-regulating heme oxygenase-1 (HO-1) expression [34].

Grape-derived ELNs are inherently biocompatible and biodegradable, remain stable under simulated gastrointestinal conditions, and enhance intestinal stem-cell proliferation [35]. They contain a water channel protein and heat shock protein 70 (HSP70) as well as abundant lipids. Research suggests that after oral administration, they stimulated the proliferation of mouse intestinal stem cells, promoted the self-renewal of intestinal epithelium through activation of the Wnt/β-catenin signaling pathway, and therefore protected mice from DSS-induced colitis [36].

Extracellular vesicles isolated from citrus sinensis can be internalized by Caco-2 cells and are suggested to regulate the expression of important genes associated with the restoration of intestinal permeability, such as *claudins* and *occludin*, or with inflammatory pathways, such as HMOX-1 and ICAM1 [51]. Nanovesicles from orange juice can also be used to treat obesity-associated intestinal complications and reverse intestinal alterations in diet-induced obese mice by increasing the villus size and modulating mRNA levels of genes involved in barrier permeability, immune response, fat absorption, and chylomicron release [52].

Lemon-derived ELNs enhanced the bile resistance of *Lactobacillus* spp. in the small intestine of mice [53]. *Clostridium difficile* (*C. diff*) is a major cause of antibiotic-associated colitis. Studies on lemon-derived ELNs showed that they elevated the levels of aryl hydrocarbon receptor (AhR) ligands, including indole-3-lactic acid (I3LA) and indole-3-carboxaldehyde (I3Alc). These metabolites induced IL-22 production and enhanced lactic-acid accumulation by suppressing *C. diff* growth and indole biosynthesis. Consequently, lemon ELNs reduced fecal shedding of *C. diff* and protected mice from infection [54].

The use of blueberry ELNs reduced TNF-α-induced reactive oxygen species (ROS) in EA.hy926 cells, reversed changes in mRNA expression of IL1RL1, IL-6, ICAM1, MAPK1, TNF-α, and TRL8, restored antioxidant cellular competence through positive regulation of HMOX1 and NRF1, and can be considered as a therapeutic vector candidate for bioactive compounds [37]. The most abundant miRNAs in blueberry ELNs mainly belong to the miR166 family and miR396 family. Results from the target gene prediction suggested that blueberry ELNs may regulate pathways associated with the human digestive system, infectious diseases, and immune system [38].

IL-1b is a potent pro-inflammatory cytokine that, together with TNF-α, induces the classical NF-kb pathway. Treatment of macrophages with ELNs isolated from apples was shown to induce a decrease in the expression of IL-1b and IL-8, suggesting a certain anti-inflammatory potential of apple ELNs [55].

Although the above research is promising, some limitations need to be pointed out. First, most conclusions are based on a single animal model (such as DSS induced colitis), which cannot fully simulate the complex immunopathological processes of human IBD. Second, it is still difficult to directly compare the results of ELNs in the existed studies, because their separation and purification methods, dosages, and administration regimens of ELNs are different. For example, the miR166 and miR396 families enriched in blueberry ELNs were reported to regulate digestive and immune pathways [38], but this was a bioinformatics prediction and lacked experimental confirmation. Future research needs to standardize the preparation and characterization of ELNs, validate them in animal models, and deeply analyze the relationship between their active ingredients (such as specific miRNAs and lipids) and specific targets, in order to promote their translation into clinical applications.

### 2.3. ELNs Isolated from Medicinal Plants

Natural compounds found in herbal plants have limited adverse effects, high therapeutic efficacy, and wide adaptability, thus playing a key role in the treatment of IBD [56]. The mechanisms of its derived ELNs are diverse, mainly focused on regulating macrophage polarization, repairing intestinal barriers, and regulating intestinal microbiota.

Pueraria lobata is a nutritious medicinal and food plant. Research indicates that ELNs derived from Pueraria lobata can induce M2 macrophage polarization from M1 macrophages, implying an anti-inflammatory effect [57]. In a DSS-induced mouse model of colitis, oral administration of different doses of ELNs derived from Pueraria Mirifica were effective in alleviating colonic shortening, and their disease activity index (DAI) scores were reduced compared to the control group. Further studies suggest that Pueraria lobata-derived ELNs may down-regulate pro-inflammatory cytokines, up-regulate barrier function mRNA expression levels, attenuate histopathological damage, improve intestinal barrier function, and selectively regulate the composition of intestinal flora [58]. Portulaca oleracea is another widely used herb. In the DSS-induced colitis in mice, oral administration of Amaranthus-derived ELNs inhibited the expression of myeloperoxidase and pro-inflammatory cytokines, and increased the level of the anti-inflammatory cytokine IL-10. They were also effective in reducing colonic shortening, and their fecal condition and colonic mucosa were superior to those of the control group. In addition, oral administration of Portulaca oleracea ELNs was colon-targeted and altered the composition and structure of the gut microbiota in colitis mice, suggesting a natural and novel potential colitis drug [59]. Taraxacum officinale, as a kind of food–medicine homologous plant, has anti-inflammation and anti-oxidation properties. Taraxacum officinale-derived ELNs can relieve hypertension in rats by acting on the intestine. There has also been research into another medicinal plant, the Taraxacum officinale. Its ELNs reduced barrier damage and local intestinal inflammation through modulating intestinal flora imbalance and short-chain fatty acid content, and therefore inhibited systemic inflammation and vascular-wall remodeling to reduce chronic intermittent hypoxia-induced hypertension [60]. Collectively, these studies indicate that medicinal plant ELNs can exert synergistic effects through multiple targets and pathways.

The mechanisms of specific active components have been further elucidated. For example, one study found that miR-7972, a major component of *fresh Rehmanniae Radix* ELNs, reduced the production of pro-inflammatory cytokines (IL-1β, IL-6, and TNF-α), ROS, and nitric oxide (NO) in LPS-exposed RAW264.7 cells, which promoted M2 macrophage polarization [39]. Additionally, this miRNA was reported to restore gut flora dysbiosis and alleviated LPS-induced lung inflammation by targeting the GPR161-mediated Hedgehog pathway.

Ginseng is a highly valuable herbal medicine containing active ingredients [61]. Ginseng-derived ELNs have been shown to decrease the levels of inflammatory cytokines such as TNF-α and IL-6 on inflammatory cells by down-regulating NF-κB expression [62]. In addition, modulation of the intestinal microenvironment by increasing the levels of probiotics (e.g., lactobacilli) could be a promising therapeutic agent for IBD. *Lycium barbarum* lipid-based edible nanoparticles inhibited the secretion of TNF-a and IL-12 and up-regulated IL-10 expression. After oral administration, these nanoparticles specifically accumulated in the inflamed colon of mice and effectively reduced ulcerative colitis [63].

The ELNs isolated from turmeric had excellent anti-inflammatory activity in vitro and in vivo, which can promote the transformation of the M1 phenotype into M2 macrophages and restore the damaged intestinal epithelial barrier [64]. Curcumin, a bioactive constituent in the ELNs from turmeric, was shown to ameliorate colitis and accelerate the regression of colitis in a mouse model by regulating the expression of pro-inflammatory cytokines, including TNF-α, IL-6, and IL-1β, as well as the antioxidant gene HO-1. Its mediated inactivation of the NF-κB pathway may contribute in part to the protective effect against colitis [65].

ELNs from edible mulberry bark were reported to offer protection against colitis in a mouse model through promoting heat shock protein family A (Hsp70) member 8-mediated activation of the AhR signaling pathway [66]. Dysbiosis of intestinal flora promoted the metastasis of breast tumors in a mouse model, whereas the consumption of edible tea flowers-derived ELNs can regulate the intestinal biota, increase the community abundance and diversity of the gastrointestinal flora, and have an inhibitory effect on the metastasis of breast tumors [67]. Tea leaves-derived ELNs can not only regulate intestinal flora, but also reduce the ROS production, inhibit the expression of pro-inflammatory factors, and increase the amount of anti-inflammatory factor IL-10 secreted by macrophages. After oral administration, they can effectively inhibit the intestinal inflammatory response and restore the broken colon barrier, thus preventing or alleviating inflammatory bowel disease [68]. Boehmeria japonica is a flowering plant native to the *Boehmeria* genus. Previous work showed that ELNs derived from Boehmeria japonica increased the production of the immunosuppressive cytokine IL-10 in dendritic cells, suggesting its ability to induce immune tolerance. In addition, these ELNs-treated DC cells exhibited a reduced immunostimulatory capacity to induce activation of Th1 and Th17 subsets, while promoting regulatory T-cell activity, which shows promise in IBD therapy [69].

In summary, compared to ELNs derived from fruits and vegetables, those from medicinal plants exhibit more diverse and complex immunomodulatory and reparative functions, demonstrating particular potential in inducing immune tolerance. It should be noted that one of the key mechanisms underlying this potential—macrophage polarization toward the M2 phenotype—exhibits a context-dependent dual role. In the inflammatory environment of IBD, M2 polarization contributes to anti-inflammatory responses and tissue repair. However, in the tumor microenvironment, M2-type tumor-associated macrophages (TAMs) may promote immunosuppression and disease progression [70]. M2-like TAMs can mediate immunosuppression by secreting anti-inflammatory cytokines (e.g., IL-10, TGF-β) and promoting regulatory T-cells recruitment, while also driving tumor progression through facilitating angiogenesis, invasion, and metastasis. Therefore, this characteristic must be carefully considered in the development of ELN-based therapies [71]. At present, research in this field still faces limitations such as reliance on single disease models and a lack of direct comparison and standardization across different ELNs. Future work should establish standardized preparation protocols, validate efficacy and long-term safety in more complex disease models, and elucidate the precise network of their multi-target synergistic actions to ultimately bridge the gap toward clinical application.

## 3. Exosomes from Edible Animal Sources for IBD Treatment

Table 1 lists the core characteristics and mechanism of action of ELNs from animal sources. Milk is a kind of food with high nutritional value. Milk-derived exosomes are suggested to play an important role in the prevention and treatment of many intestinal diseases by regulating intestinal immune homeostasis, restoring intestinal flora composition, and improving intestinal structural integrity [40]. In a typical study, two subpopulations of extracellular vesicles (P35K EV and P100K EV) were isolated from commercial milk using ultracentrifugation at 35,000 *g* and 100,000 *g*, respectively. The effects of these two different subpopulations of extracellular vesicles on colitis may be complementary. Both vesicles modulated the intestinal microbiota, restored intestinal impermeability, and supplemented mucin secretion, with ameliorative effects on DSS-induced colitis. Moreover, P35K EV regulated inflammation by promoting immune-cell proliferation, differentiation, and survival, while P100K EV resolved inflammation by restoring normal levels of immune-, inflammatory-, and colitis-associated microRNAs [41]. In another study, RNA sequencing and proteomic analysis revealed that abundant proteins and microRNAs in milk exosomes took part in the regulation of immune and inflammatory pathways. They can prevent colonic shortening and reduce intestinal epithelial rupture by inhibiting the NLRP3 inflammasome activation and the TLR4-NF-κB signaling pathway, and restoring the Treg/Th17 cellular homeostasis and intestinal microbiota [42]. Similarly, oral administration of milk exosomes effectively attenuated the symptoms of DSS-induced colitis in mice by suppressing the expression of chemokines (CXCL1, CXCL2, and CXCL3) and pro-inflammatory cytokines (IL-1β and IL-6), and promoting the expression of anti-inflammatory genes [43]. Na/K + ATPase is a major regulator of sodium and water movement in the colon, and patients with diarrhea in IBD are partly attributed to the reduced electrolyte and water absorption. Experimental results indicated that milk exosomes could potentiate Na/K + ATPase activity in Caco-2 cells via EP3 and EP4 receptors [44].

Dysbiosis of the intestinal microflora is closely associated with the development of colitis. Milk exosomes have been shown to optimize the gut microbiota and manipulate intestinal gene expression to alleviate colitis [72]. In addition, oral administration of milk exosomes was found to promote the utilization of n-3 polyunsaturated fatty acids, such as eicosapentaenoic acid and docosahexaenoic acid, in the colonic tissues of mice with colitis and reversed inflammation-induced higher concentrations of certain amino acids in the tissues and feces, suggesting their potential to restore metabolic disturbances caused by inflammation [73].

One study reported that the number of exosomes in yak milk is 3.7 times higher than that in cow milk. Under hypoxic conditions, yak milk exosomes significantly promoted the expression of oxygen-sensitive prolyl hydroxylase-1 and decreased the expression of hypoxia-inducible factor-alpha and the associated downstream target vascular endothelial growth factor in IEC-6 cells, which in turn promoted the intestinal epithelial cell survival [45]. Proteomics revealed differential expression of proteins in yak milk versus milk exosomes and identified the protein CD46 as a regulator of IEC-6 inflammatory injury [46]. In another study, yak-milk exosomes activated the PI3K/AKT/C3 signaling pathway more than cow-milk exosomes to reduce the incidence and severity of intestinal inflammation. Goat milk also has a positive effect on IBD. Colostrum exosomes (C-Exo) and mature-milk exosomes (M-Exo) were isolated from goat milk. It was found that the both of them can alleviate intestinal inflammation by decreasing LPS-induced pro-inflammatory cytokine release, inhibiting the increase in NLRP3 protein and activation of the TLR4/NF-κB signaling pathway, while the inhibitory effect of colostrum exosomes was more obvious [74].

Eggs and royal jelly are other common animal-derived foods with high nutritional value. Exosomes from their sources may play a positive role in IBD. For example, albumen exosomes could alleviate LPS-induced inflammation in IPEC-J2 cells, in which specific miR-22 could alleviate pro-inflammatory cytokine overexpression by inhibiting NF-κB and p53 pathways. Additionally, miR-22 targeted ATM, the upstream gene of Tp53, to inhibit LPS-induced apoptosis, suggesting that albumen exosomes have therapeutic potential in intestinal inflammation [47]. It has been shown that royal jelly had a significant therapeutic effect on rats with acetic acid-induced colitis, reducing the number of mast cells and the degree of colonic erosion [75]. The royal jelly-derived exosomes had good antimicrobial properties, with bacteriostatic, bactericidal, and biofilm inhibitory effects against *Staphylococcus aureus* [76].

## 4. Naturally Occurring Dietary ELNs as Delivery Systems for IBD Treatment

**Figure 2:**
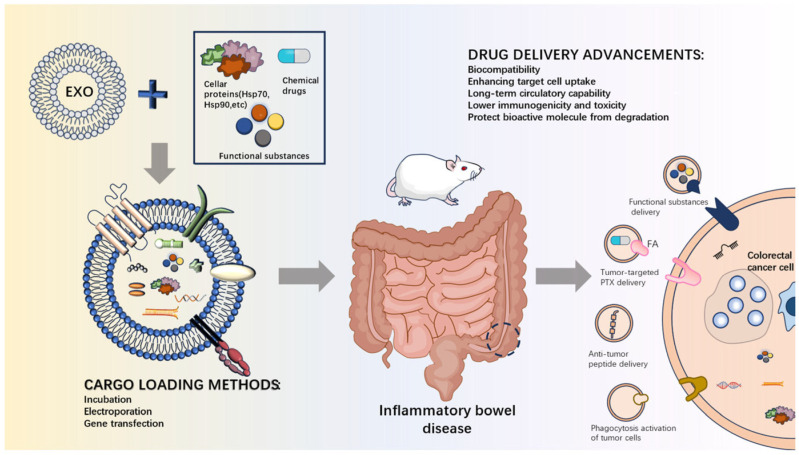
Schematic of engineered ELNs as a delivery system for IBD therapy, illustrating the primary techniques for loading therapeutic molecules, the delivery performance, and the oral administration route to colorectal cancer cells.

ELNs have many advantages as delivery systems (Figure 2). The lipid bilayer of ELNs can encapsulate a variety of physiologically active substances and protect them from degradation [77]. Additionally, ELNs can cross biological barriers and selectively target specific cells or tissues. For example, milk-derived ELNs can survive in the highly acidic environment of the stomach and gut and cross the biological barrier to reach the target tissue, making it an excellent vehicle for oral delivery [78]. In contrast to ELNs of mammalian origin, dietary plants are free of zoonotic or human pathogens and have an excellent safety profile and cost-effective production [79]. Table 2 lists some recent examples on the utilization of ELNs as delivery systems for IBD treatment.

Curcumin is a naturally occurring polyphenol with low bioavailability due to its hydrophobicity. Encapsulation in milk-derived ELNs increased the stability of curcumin during in vitro digestion. Caco-2 cells also showed that the curcumin-loaded ELNs could cross the intestinal barrier and enter the circulation [80]. Another work employed three methods (direct loading, sonication, and extrusion) to encapsulate curcumin in the tomato-derived ELNs and investigated the anti-inflammatory activity of curcumin by a LPS-induced THP-1 cell line. It was found that curcumin-loaded ELNs showed anti-inflammatory activity by decreasing the mRNA levels of IL-6 and IL-β, and the sonicated ELNs showed the most pronounced anti-inflammatory effect [81].

Astaxanthin is a red-pigmented carotenoid with a wide range of anti-inflammatory, antioxidant, anti-apoptotic, and anti-proliferative properties [82]. It is a potential candidate for the treatment of chronic inflammatory diseases [83]. Astaxanthin was loaded into hyaluronic acid-modified milk ELNs using sonication, and the targeting of this delivery system to inflammatory cells was significantly enhanced. In a lipopolysaccharide-induced cellular model, the system enhanced the cellular uptake of astaxanthin compared to free astaxanthin and significantly alleviated the overproduction of reactive oxygen species and depolarization of mitochondrial membrane potential. The expression of IL-1β and IL-6 were also inhibited, suggesting a potential application for the prevention of chronic inflammatory diseases [84].

α-mangostin is a natural flavonoid with demonstrated antimicrobial and anti-inflammatory properties. A recent study has shown that when loaded into milk ELNs, the antibacterial efficiency of α-mangostin was enhanced, which can remove 99% of bacteria from macrophages [85]. In addition, the milk ELNs loaded with α-mangostin exhibited high mucus permeability and improved antimicrobial capacity.

Luteolins are polyphenolic compounds widely found in plants such as chili peppers and cabbage. They have antioxidant, anti-inflammatory, anti-tumor, and antidiabetic properties. ELNs isolated from plant sesame leaves had a high encapsulation rate for luteolins, and their interactions were mainly van der Waals forces and hydrogen bonding. Compared to free luteolin, ELNs-encapsulated luteolin demonstrated significantly enhanced bioavailability and stability after simulated digestion, showed better performance in terms of cellular uptake and antioxidant and anti-inflammatory activities [86].

**Table 2:** Utilization of ELNs as delivery systems for IBD treatment.

ELNs Origin	Bioactive Compound	Results	Ref.
Milk-derived exosomes	Curcumin	ELNs increased the stability of curcumin during in vitro digestion and the ability to cross the intestinal barrier	[80]
Tomato fruit-derived ELNs	Curcumin	Curcumin-loaded vesicles exhibit anti-inflammatory activity by reducing IL-6 and IL-β mRNA levels.	[81]
Milk-derived exosomes	Astaxanthin	Exosomes target inflammatory cells, increasing astaxanthin uptake, reducing excessive production of ROS, and lowering inflammatory responses.	[84]
Milk-derived exosomes	α-mangostin	Milk exosomes loaded with α-mangostin exhibit high mucus permeability, enhancing antimicrobial capacity and eliminating 99% of bacteria in macrophages.	[85]
Plant sesame leaves-derived ELNs	Luteolins	ELNs encapsulation of luteolin significantly improves its bioavailability and stability, and exhibits better performance in terms of cellular uptake and antioxidant and anti-inflammatory activity.	[86]

## 5. Modification of ELNs for IBD Treatment

ELNs of dietary animal- and plant origin have intrinsic therapeutic activity and are good delivery systems that can transfer functional substances to receptor cells to mediate intercellular communication. However, the ELNs often exhibit poor therapeutic effects due to low targeting efficiency, and thus engineering ELNs has now become a trend to improve targeting.

The targeting ability can be achieved by modifying the surface of ELNs with ligands such as folic acid (FA) that can bind to cell-specific receptors. High-affinity folate receptor (FR) expression is elevated in many human tumors, and in a mouse model of colon cancer, grapefruit-derived nanoparticles conjugated with FA improved tumor targeting. Similarly, encapsulating the chemotherapeutic drug paclitaxel within these FA-modified nanoparticles did not compromise their FA-mediated target delivery [87]. In another work, the doxorubicin-loaded ginger-derived nanoparticles are modified by FA, aiming to increase tumor efficacy. Experimental data showed that the modification increased the residence time of the ELNs at the tumor site and, meanwhile, increased their penetration in the tumor tissue [88]. Overexpression of CD98 on the surface of colonic epithelial cells and macrophages may induce the onset and progression of IBD. In this regard, the siCD98-loaded nanoparticles were treated by surface modification of CD98 antibodies. The nanoparticles were then incorporated in the chitosan/alginate hydrogels. It was found that they can reduce the colonic CD98 expression and attenuate the severity of colitis after oral administration to mice [89].

Fusion with other cell membranes with specialized receptors in the ELNs membrane is another effective way to enhance the specific targeting of ELNs. In a previous study, the grapefruit-derived ELNs loading curcumin were coated with leukocyte membranes enriched with inflammatory receptors. In a DSS-induced colitis model, mice injected intravenously with these ELNs showed improved symptoms of colonic inflammation, which was mainly attributed to the activated leukocyte membranes that allowed the ELNs to reach the precise location of the inflammation site [90].

## 6. Prospects and Challenges

Dietary-derived ELNs, owing to their natural origin, biocompatibility, and targeting ability, have become a research topic of great interest in IBD therapy. ELNs can regulate gene expression, cellular processes, and signaling pathways in target cells. They also play a role in intercellular communication, thereby affecting physiological processes and immune responses. Despite the multiple positive effects currently shown for dietary sources of ELNs in disease treatment, however, there are still some challenges in the practical application of IBD therapy.

The first is the isolation and extraction phase of ELNs. For ELNs of mammalian origin, the identification of specific surface markers, such as the four-transmembrane protein superfamily and endosomal sorting complex required for transport complex-associated proteins, helps to specifically identify the isolated ELNs. However, the ELNs of plant origin do not have established systematic marker proteins, which brings some obstacles to the purity of isolation and identification. Therefore, there is a need to pay more attention to the biological characteristics of ELNs and adopt more reliable analytical methods to identify ELNs. The second is the preservation of ELNs. ELNs are more stable when stored at −80 °C, but this temperature is not favorable for transportation and handling. The use of freeze-drying technology and the addition of a cryoprotectant such as alginate have been developed to allow ELNs to be stored at room temperature. The modification of ELNs to make them more stable could be a future research direction. The third is that more experiments are needed to further investigate the mechanism of ELNs on prevention and amelioration of IBD, such as the role of ELNs in the modulation mechanism to the host’s immune system and gut flora. The fourth is that the surface modification can improve their stability and targeting ability of ELNs; however, the interaction mechanisms between modified materials and ELNs remain unknown. Finally, despite the potential of dietary-derived ELNs in treating IBD, their translation into clinical applications still faces regulatory and safety assessment challenges. The complexity of ELNs lies not only in their nanoscale size but also in the diverse food-matrix components from which they are derived. Therefore, it is imperative to systematically investigate the safety of long-term consumption, potential immunogenicity, and long-term effects on gut microbiota. There is also an urgent need to establish standard assessment methods tailored to the characteristics of ELNs. Furthermore, the dietary ELNs products face the ambiguous classification issue between “dietary supplements” and “drugs” in terms of regulation. This necessitates clarifying their regulatory pathway as a prerequisite for product development.

## 7. Conclusions

ELNs isolated from dietary sources have the advantage of being abundant and accessible in large quantities. ELNs contain many natural components with antioxidant, anti-inflammatory, and immunomodulatory functions, and the interaction of natural and exogenous components increases the therapeutic efficacy for IBD. Additionally, their lipid bilayer structure and good biocompatibility make them suitable as bioactive delivery systems. Surface modification may further improve the colonic targeting of ELNs. The positive effects of dietary-derived ELNs on IBD include (i) regulating the balance of pro-inflammatory and anti-inflammatory cytokines to mitigate the progression of inflammation by balancing the polarization of M1/M2 macrophages and by inhibiting the pathways downstream of the activation of NLRP3 inflammatory ELNs such as those derived from vegetable sources of ginger, garlic, and shiitake mushrooms as well as herbal sources; (ii) repairing the intestinal barrier and reducing intestinal-barrier permeability by promoting the survival and proliferation of intestinal epithelial cells and the expression of tight junction proteins; (iii) remodeling the gut microbial environment by increasing the abundance and diversity of gut microbes, increasing the probiotic number, and inhibiting the growth of harmful bacteria.

## Figures and Tables

**Table 1:** Summary of core characteristics and mechanism of action of ELNs from edible sources.

ELNs Origin	Core Loaded Cargo	Disease Model	Key Mechanisms of Action	Refs.
Ginger	Specific miRNAs (Osa-miR164d, mdo-miR7267-3p), gingerol analogs, volatile oils	DSS-induced mouse colitis model	Inhibit NLRP3 inflammasome and NF-κB pathway, induce M2 macrophage polarization, regulate gut microbiota metabolism, repair intestinal mucosal barrier	[23,24,25,26,27]
Garlic	Specific miRNAs (peu-MIR2916-p3, Han-miR3630-5p)	DSS-induced mouse colitis model	Inhibit NLRP3 and TLR4/NF-κB pathway, regulate the growth of intestinal commensal bacteria, protect colonic barrier	[28,29,30]
Broccoli	Sulforaphane	Dendritic cell in vitro model	Target AMPK pathway in dendritic cells, regulate intestinal immune homeostasis	[31]
Kidney bean	Short-chain fatty acids;	Rat obesity model	Modulate gut microbiota composition, increase SCFA levels, improve obesity phenotype	[32]
Grapefruit	Phosphatidylethanolamine, phosphatidylcholine	IL-10 knockout mouse spontaneous colitis model, DSS-induced mouse colitis model	Activate Nrf2 and Wnt/TCF4 pathways, inhibit pro-inflammatory cytokines, upregulate HO-1 expression, ameliorate colitis	[33,34]
Grape	Aquaporins, heat shock protein 70; abundant lipids	DSS-induced mouse colitis model	Activate Wnt/β-catenin pathway, promote intestinal stem cell proliferation and intestinal epithelial self-renewal	[35,36]
Blueberry	miR166 family, miR396 family	EA.hy926 cell inflammation model	Reduce ROS production, regulate inflammation and antioxidant-related genes, modulate digestion and immune-related pathways	[37,38]
Fresh Rehmannia Glutinosa	MmiR-7972	LPS-stimulated RAW264.7 cell inflammation model, LPS-induced mouse lung inflammation model	Inhibit pro-inflammatory factors and ROS/NO production; promote M2 macrophage polarization; target Hedgehog pathway; restore gut microbiota balance	[39]
Cow’s milk	Immune and inflammation-related microRNAs	DSS-induced mouse colitis model	Regulate gut microbiota and barrier function, inhibit NLRP3 and TLR4/NF-κB pathway, restore immune cell homeostasis, improve electrolyte absorption and metabolic disorders	[40,41,42,43,44]
Yak’s milk	CD46	Hypoxia-induced IEC-6 cell injury model, intestinal inflammation model	Regulate hypoxia-related gene expression, protect intestinal epithelial cells, regulate inflammatory injury via CD46, activate PI3K/AKT/C3 pathway, alleviate inflammation	[45,46]
Egg white	Specific miR-22	LPS-induced IPEC-J2 cell inflammation model	Inhibit NF-κB and p53 pathways, target ATM gene, reduce pro-inflammatory cytokine overexpression and cell apoptosis	[47]

## Data Availability

No new data were created or analyzed in this study. Data sharing is not applicable to this article.
